# Refining the diagnosis of Huntington disease: the PREDICT-HD study

**DOI:** 10.3389/fnagi.2013.00012

**Published:** 2013-04-02

**Authors:** Kevin M. Biglan, Ying Zhang, Jeffrey D. Long, Michael Geschwind, Gail A. Kang, Annie Killoran, Wenjing Lu, Elizabeth McCusker, James A. Mills, Lynn A. Raymond, Claudia Testa, Joanne Wojcieszek, Jane S. Paulsen, and the PREDICT-HD Investigators of the Huntington Study Group

**Affiliations:** ^1^Department of Neurology, University of RochesterRochester, NY, USA; ^2^Department Biostatistics, University of IowaIowa City, IA, USA; ^3^Department of Psychiatry, University of IowaIowa City, IA, USA; ^4^Department of Neurology, University of California San FranciscoSan Francisco, CA, USA; ^5^Department of Neurology, Westmead HospitalSydney, NSW, Australia; ^6^Division of Psychiatry, University of British ColumbiaVancouver, BC, Canada; ^7^VCU Parkinson's and Movement Disorders Center Virginia Commonwealth UniversityRichmond, VA, USA; ^8^Department of Neurology, Indiana UniversityIndianapolis, IN, USA

**Keywords:** Huntington's disease, trinucleotide repeat diseases, cohort studies, natural history studies, outcome research

## Abstract

Participants with the gene expansion for Huntington disease (HD) but not yet diagnosed were evaluated annually. Unidimensional diagnosis (UD) was a motor diagnosis defined as a diagnostic confidence level (DCL) of 4 (unequivocal motor signs, ≥99% confidence) on the standardized motor exam of the Unified Huntington Disease Rating Scale (UHDRS). Multidimensional diagnosis (MD) was defined as answering yes on Question 80 (Q80) of the UHDRS, ≥99% confidence of manifest HD based on the *entire* UHDRS. Motor, cognitive, and behavioral measures of phenotype at first diagnosis were compared by t-tests between participants diagnosed via motor exam (UD) and those diagnosed via multidimensional input (MD). Cluster analysis identified clusters based on UHDRS domains.186 participants received a diagnosis of HD during a maximum of 6.4 years of follow-up. In 108 (58.1%) the diagnosis by MD and UD occurred simultaneously, while in 69 (37.1%) the diagnosis by MD occurred prior to UD. Participants who were diagnosed by MD prior to UD were less impaired on motor (12.2 ± 6.7 vs. 22.4 ± 9.3, *p* < 0.0001), and cognitive (290.7 ± 56.2 vs. 258.0 ± 53.7, *p* = 0.0002), but not behavioral measures (16.3 ± 21.2 vs. 18.6 ± 22.1, *p* = 0.49) when compared with those diagnosed simultaneously. Cluster analysis identified three clusters that represented primarily cognitively impaired, behaviorally impaired, and cognitively preserved phenotypes. A multidimensional method results in an earlier diagnosis with less motor and cognitive impairment than a motor diagnosis. Findings have implications for designing preventive trials and providing clinical care in prodromal HD.

## Introduction

Huntington disease (HD) is an adult-onset, autosomal dominant, progressive, and fatal neurodegenerative disease characterized by the clinical triad of a movement disorder, cognitive decline, and behavioral disturbances caused by a cytosine-adenine-guanine (CAG) repeat in the 5′-translated region of the gene on the short arm of chromosome 4 (Duyao et al., 1993). The precise point of disease diagnosis is poorly characterized, with clinical abnormalities emerging gradually over many years during a “pre-manifest” or prodromal phase (Huntington Study Group, 2006; Paulsen et al., 2006).

A challenge of therapeutic research is in the identification of treatments that impact the manifestation of disease in individuals at varying stages of disease progression. For the neurodegenerative diseases, much effort has been devoted to early identification and staging using clinical outcome measures or biomarkers. For instance, there are widespread efforts to detect “mild cognitive impairment” prior to dementia so that therapeutics might be considered before extensive cell death has occurred. Even in HD, in which a cohort can be identified years prior to diagnosis, challenges remain in designing trials aimed at delaying illness progression. The Neurobiological Predictors of Huntington's Disease (PREDICT-HD) study is a longitudinal prospective evaluation in individuals at risk for HD with known gene status. The PREDICT-HD study should help identify outcomes for use in trials aimed at delaying the manifestation of illness in prodromal HD. However, in order to show that an intervention can delay disease, there needs to be consensus on how to best define the clinical diagnosis of HD. The traditional method of HD diagnosis rests on the motor manifestation of disease though the cognitive and psychiatric aspects of HD have been recognized for decades. Efforts toward more refined disease staging may be improved with a more comprehensive consideration of HD. Therefore, we compared two methods of diagnosis in the PREDICT-HD cohort: a multidimensional diagnosis (MD) and a unidimensional diagnosis (UD) or motor diagnosis.

## Materials and methods

All aspects of the study were approved by the Institutional Review Board at each participating institution. Participants signed consents for participation and to release their de-identified data for analyses.

### Overview of PREDICT-HD

The PREDICT-HD study is designed to prospectively characterize refined clinical, neurobiological, and neurobehavioral markers of HD prior to the point of traditional motor diagnosis in a population known to carry the HD CAG expansion (Paulsen et al., 2006). Participants at risk for HD were recruited from 32 sites in the United States, Canada, Australia, and Europe beginning in 2001. All participants were required to have voluntarily undergone genetic testing for the HD CAG expansion independent from the study. Participants were evaluated annually with standardized assessments of motor, cognition, behavior, function, and clinical diagnosis.

Only individuals with the HD CAG expansion and without manifest disease (prodromal HD) as defined by the absence of unequivocal motor signs (diagnostic confidence level of less than 4 on question 17 of the UHDRS, Table 1A) on their initial examination were included in the current analysis. Control subjects were those participants who had tested negative for the HD CAG expansion and had participated in at least two visits. For purposes of this analysis, the last visit in controls was used for comparison with cases.

**Table 1:** **The Unified Huntington Disease Rating Scale diagnostic confidence level and Q80 diagnostic criteria**.

**A. Diagnostic confidence level**
**To what degree are you confident that this person meets the operational definition of the unequivocal presence of an otherwise unexplained extrapyramidal movement disorder (e.g., chorea**, **dystonia, bradykinesia, rigidity) in a subject at risk for HD?**
0 = normal (no abnormalities)
1 = non-specific motor abnormalities (less than 50% confidence)
2 = motor abnormalities that may be signs of HD (50–89% confidence)
3 = motor abnormalities that are likely signs of HD (90–98% confidence)
**4 = motor abnormalities that are unequivocal signs of HD (≥99% confidence)**
**B. Q80 diagnostic criteria**
**Based on the entire UHDRS (Motor, Cognitive, Behavioral, and Functional components) do you believe with a confidence level ≥99% that this participant has manifest HD? (0 = No, 1 = Yes)**

### Clinical assessments

#### Huntington disease clinical diagnosis

Motor Diagnosis: The Huntington Disease Rating Scale (UHDRS) diagnostic confidence level (DCL) is the standard measure used for clinical diagnosis in at-risk individuals and is based solely on the motor evaluation. It is a categorical scale (Table 1A) with a range from 0 (normal) to 4 (unequivocal signs of HD, ≥99% confidence ≥ on the part of the examiner). The DCL has previously shown fair inter-rater reliability (weighted kappa = 0.67, SE = 0.09) (Hogarth et al., 2005). Participants had a clinical diagnosis of HD at the time of the first rating of a DCL = 4.

Multidimensional Diagnosis: Question 80 (Q80) of the UHDRS asks raters to take into account all aspects of the UHDRS (motor, cognitive, behavioral, and functional) and to make a decision (yes or no) whether a subject has a diagnosis of HD with a confidence level 99% (Table 1B). The first occurrence of Q80 = yes was the multidimensional diagnostic criteria used for the current analyses.

The primary analysis compared participants who were diagnosed by MD prior to receiving a diagnosis by UD with participants who received a diagnosis of MD and UD simultaneously. A small proportion of individuals received a diagnosis by UD prior to MD and these participants were not included in the analysis.

### Unified huntington disease rating scale outcomes

The current analyses focused solely on the UHDRS assessments, since UD is rated on the motor UHDRS only and MD specifically asks raters to make a determination based on the entirety of the Hungtington Study Group (1996). The motor UHDRS assessed for the presence and severity of motor features (Hungtington Study Group, 1996). The motor UHDRS is a standardized assessment consisting of 31 items rated on a scale from 0 to 4 with a score of 0 indicating no abnormalities and 4 indicating the most severe impairment. The maximum possible total score is 124. Previously motor scores have been shown to distinguish controls from prodromal HD cases and subtle motor abnormalities were associated with closer estimated diagnosis of disease (Biglan et al., 2009). In manifest HD, oculomotor, rigidity, chorea, dystonia, and bradykinesia domains have been identified and were used to clarify if specific motor features were associated with specific clusters at time of clinical diagnosis (Marder et al., 2000).

The cognitive section of the UHDRS includes verbal fluency, symbol digit modalities test, and Stroop word, color, and interference tests (Hungtington Study Group, 1996; Biglan et al., 2009). Each of these cognitive tests has been shown to distinguish gene mutation carriers from controls in prodromal HD (Paulsen et al., 2008; Stout et al., 2011). Total cognitive scores are calculated by summing the five individual scores in the UHDRS cognitive domain.

The behavioral section of the UHDRS consists of 11 items evaluating various behavioral signs and symptoms. Individuals are ranked on both severity and frequency on a 0 to 4 scale with 0 being not present and 4 being severe and frequent (Hungtington Study Group, 1996). Total behavioral scores are calculated by summing the severity and frequency items and ranges from 0 (no behavioral symptoms) to 88 (most severe behavioral symptoms).

The functional section of the UHDRS includes the Functional Assessment Scale, Independence Scale, and the Total Functional Capacity (TFC) (Hungtington Study Group, 1996). The TFC is a standard assessment of overall function in HD and has a demonstrated reliability for indexing progression in various diagnosed HD populations (Marder et al., 2000; Huntington Study Group, 2001). The TFC rates individuals' function on the following domains: occupation, handling finances, domestic chores, and activities of daily living. The TFC ranges from 13 (normal function) to 0 (complete loss of function). In prodromal HD, there is a strong tendency for participants to have the maximum score, as most have normal function; thus the TFC was treated as a dichotomous variable (TFC < 13) to indicate whether an individual has some kind of impairment in functionality for daily living (Paulsen et al., 2010). For assessment of employment status UHDRS item #43 (ability to work at accustomed employment) and item #44 (ability to work at any employment) were used.

### Statistical analysis

Kaplan-Meier curves were generated to describe the probability of being diagnosis-free over time and to evaluate the temporal relationship between incident diagnoses using the different diagnostic criteria.

UHDRS total motor, total cognitive, and total behavioral scores at the time of incident diagnosis were compared between the different diagnostic groups using *t*-tests.

Chi-square tests were used to compare differences in the frequency of diagnosis by the same vs. different raters between the different diagnostic groups.

To evaluate the factors associated with diagnosis in those participants who received a diagnosis of MD prior to UD, K-mean clustering with the pseudo-F statistic criterion was performed to identify categories of participants (clusters) at the time of diagnosis. The UHDRS total motor score, total cognitive score, and total behavior score at diagnosis were used in the cluster analysis. In order to ascertain if raters utilized participants' functional status in the diagnostic decision, TFC, and employment status were compared across the clusters. To determine if specific motor features were associated with different clusters, the sum of each motor domain was compared across clusters. The ANOVA, Fisher Exact Tests, or Kruskall-Wallis Test were performed as appropriate to determine the difference between the clusters and the control group and *post-hoc* pairwise comparisons using *t*-tests or chi-square tests, corrected for multiple comparisons (alpha < 0.01) to determine the statistical ordering among the groups.

## Results

Since 2001, a total of 1054 individuals have been enrolled in the PREDICT-HD study. Of these participants, 821 (78%) carried the CAG expansion and were considered prodromal (DCL < 4) at baseline. A total of 233 (22%) of the participants enrolled did not carry the CAG expansion (controls); of these, 194 had at least two follow-up visits.

**Figure 1:**
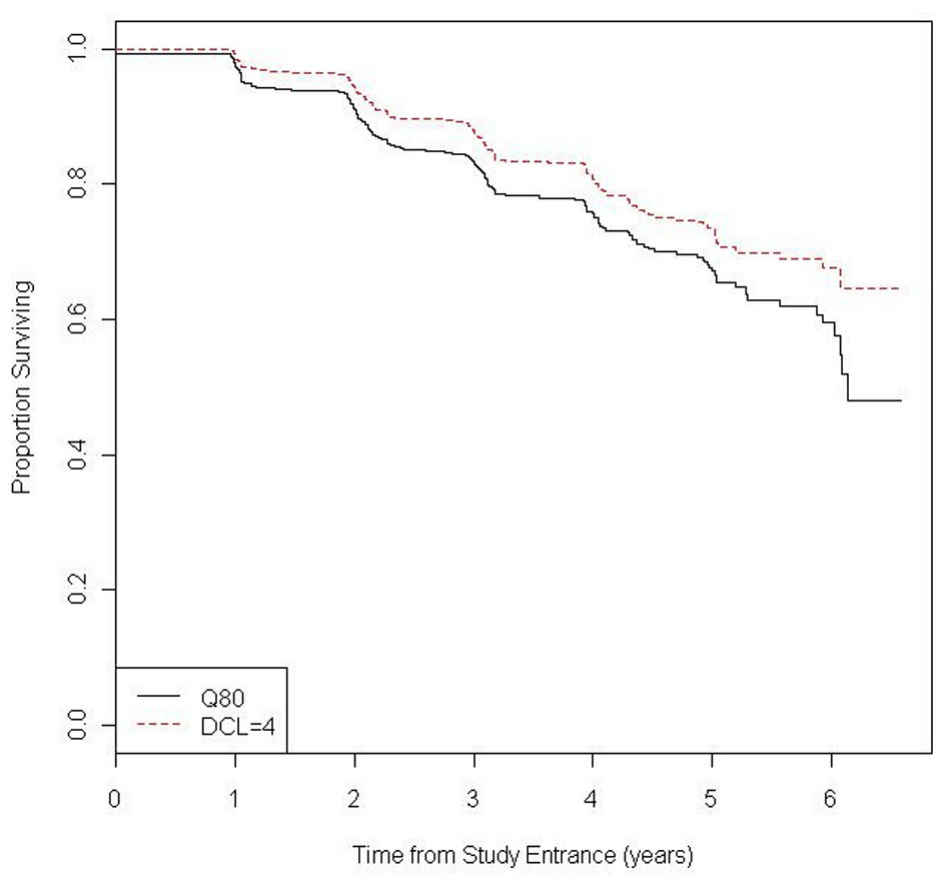
**Kaplan-Meier estimate of the probability of being diagnosis-free during follow-up by type of diagnosis (UHDRS Q80 = yes and UHDRS DCL = 4)**.

Over a mean follow-up of 3.1 years (SD = 1.4 years and range = 6.4 years) a total of 186 CAG expanded participants (23% of total CAG expanded) received a first diagnosis of manifest HD by either diagnostic criteria (MD or UD). Of these diagnosed individuals, 108 (58.1%) received a diagnosis by UD and MD simultaneously, 69 (37.1%) by MD prior to UD, and 9 (4.8%) by UD prior to MD. Figure 1 shows the Kaplan-Meier estimates of the diagnosis-free probability curves over 6 years of follow-up for diagnosis based on the UD and MD criteria.

Of those diagnosed, 148 (79.6%) had the same rater, whereas 38 (20.4%) had different raters for UD and MD. DCL = 4 and Q80 diagnoses were more likely to occur simultaneously when the rater was the same (89.8%). Q80 diagnosis also preceded DCL = 4 diagnosis more often when the rater was the same (73.9%) (Table 2).

**Table 2:** Diagnostic agreement between by same vs. different raters^*^^,^^†^.

**Diagnosis**	**Same rater**	**Different rater**	**Total**
Simultaneous Q80/DCL = 4	97 (89.8%)	11 (10.2%)	108
Q80 before DCL = 4	51 (73.9%)	18 (26.1%)	69
Total	148 (83.6%)	29 (16.4%)	177

* p = 0.005 for the comparison of clinical diagnosis by rater category.

† Does not include the 9 participants where DCL = 4 occurred before Q80.

Table 3 demonstrates the clinical features at the time of diagnosis by the different criteria. Participants who were diagnosed by MD prior to UD were less impaired on UHDRS total motor scores (12.2 ± 6.7 vs. 22.4 ± 9.3, *p* < 0.0001) and on total cognitive scores (290.7 ± 56.2 vs. 258.0 ± 53.7, *p* = 0.0002) compared with individuals who received the diagnoses simultaneously. There was no statistical difference on UHDRS behavioral scores (16.3 ± 21.2 vs. 18.6 ± 22.1, *p* = 0.49) between the two groups.

**Table 3:** **Clinical features at time of diagnosis**.

**Variables**	**Q80 before DCL = 4 (*n* = 69)**	**Simultaneous Q80/DCL = 4 (*n* = 108)**	**Controls^†^ (*n* = 194)**	*p*-value^*^
Gender (%F)	65.7	66.7	66.0	0.89
Age (mean ± SD)	46.3 ± 9.2	46.7 ± 10.3	46.7 ± 11.1	0.81
CAG (mean ± SD)	43.1 ± 3.1	43.5 ± 3.1	20.1 ± 3.5	0.43
UHDRS motor (mean ± SD)	12.2 ± 6.7	22.4 ± 9.3	2.8 ± 3.1	<0.001
UHDRS cognition (mean ± SD)	290.5 ± 56.5	258.0 ± 53.7	341.4 ± 47.4	<0.001
UHDRS behavior (mean ± SD)	16.3 ± 21.2	18.6 ± 22.1	5.7 ± 9.3	0.49
UHDRS TFC (%<13)	31.9	48.1	7.0	0.03

* p-values are for the comparison between Q80 diagnosis before DCL = 4 and simultaneous diagnosis.

† The values of controls were taken at the last visit.

A cluster analysis using *K*-mean clustering in the participants that received MD prior to UD was performed and a three cluster solution was identified based on the pseudo-*F* statistic criterion. Table 4 shows the mean total motor, cognitive, and behavioral scores by clusters and controls. Cluster 1 identifies a predominantly cognitively impaired phenotype because it had the lowest UHDRS cognitive mean, but the second highest behavior mean and the highest total motor mean (Cluster 1 might also be labeled as predominantly cognitive/motor). Cluster 2 identifies a predominantly behaviorally impaired phenotype as it had the highest behavior mean, but had the second highest cognitive mean and the lowest total motor mean among the gene-expanded participants. Cluster 3 represents a cognitively preserved group because the cluster had the highest cognitive mean (even higher than controls), the lowest behavior mean, and the second highest total motor mean among gene-expanded participants. All clusters had significantly worse motor scores compared with controls. A more detailed assessment of motor features using the motor sub-domains (Table 4) suggests that cluster 3 (cognitively preserved) had the most chorea while cluster 1 (cognitively impaired) performed the worst on the bradykinesia domain. A cluster analysis of the participants that received simultaneous diagnoses identified a three-cluster solution that was qualitatively similar (i.e., cognitive, behavioral, and preserved phenotypes), except that participants performed worse on motor and cognitive measures than the same clusters in participants with MD prior to UD (results not shown). All three clusters were more likely than controls to have greater functional impairment as measured by TFC. Whereas participants in cluster 3 were more likely to be employable compared to the other clusters, this did not meet the threshold for significance and all clusters were less likely to be employable compared with controls (see Supplementary Table e-1).

**Table 4:** **Group comparisons between clusters**.

**Variables**	**Control (*n* = 194)**	**Cluster 1 (*n* = 21)**	**Cluster 2 (*n* = 32)**	**Cluster 3 (*n* = 15)**	**ANOVA *p*-value**	**Pair-wise comparisons (alpha 0.01)**
Age (mean ± SD)	46.75 ± 11.13	45.70 ± 9.32	47.26 ± 9.97	44.70 ± 7.70	0.85	–
CAG (mean ± SD)	20.12 ± 3.45	43.58 ± 2.59	43.22 ± 3.66	42.73 ± 2.09	<0.001	Control < C1, C2, C3
**UHDRS**						
Total motor						
(mean ± SD)	2.75 ± 3.08	16.52 ± 5.60	9.56 ± 6.03	11.86 ± 6.78	<0.001	Control < C2, C3 < C1
Motor domains						
Oculo (mean ± SD)	0.65 ± 1.23	4.52 ± 2.36	2.34 ± 2.89	3.57 ± 3.06	<0.001	Control < C2 < C1; Control < C3
Brady (mean ± SD)	1.44 ± 1.91	6.67 ± 3.12	4.03 ± 3.10	3.79 ± 2.81	<0.001	Control < C2, C3 < C1
Rigidity (mean ± SD)	0.31 ± 0.68	0.67 ± 1.15	0.53 ± 0.72	0.92 ± 1.07	0.004	Control < C3
Dystonia (mean ± SD)	0.06 ± 0.34	1.05 ± 1.56	0.38 ± 0.87	0.43 ± 0.76	<0.001	Control < C2 < C1; C3 < C1
Chorea (mean ± SD)	0.29 ± 0.69	3.62 ± 2.52	2.28 ± 2.05	3.14 ± 2.28	<0.001	Control < C1, C3; C2 < C1
Cognition (mean ± SD)	341.4 ± 47.4	224.0 ± 25.7	303.8 ± 19.4	361.8 ± 22.6	<0.001	C1 < C2 < Control, C3
Behavior (mean ± SD)	5.69 ± 9.29	12.65 ± 18.63	20.25 ± 21.28	7.47 ± 10.05	<0.001	Control, C3 < C2

Cluster 1, predominantly cognitive; Cluster 2, predominantly behavioral; Cluster 3, cognitively preserved.

There was no statistical difference between the clusters in the proportion of raters that were the same vs. raters who were different (see Supplementary Table e-2).

## Discussion

In participants with prodromal HD enrolled in the PREDICT-HD study, a multidimensional diagnosis occurs earlier and with less motor and cognitive impairment than a diagnosis based on the motor examination. Given the results of our analysis, a diagnosis that considers cognitive and behavioral features in addition to motor features has face validity. Therefore, compared to the traditional motor diagnosis, a multidimensional diagnosis may be a preferable outcome for use in future trials aimed at delaying the manifestation of HD.

The current analysis also identified different phenotypes in HD at the time of diagnosis: predominantly cognitively impaired (with motor impairments), predominantly behaviorally impaired, and cognitively preserved. These phenotypic clusters had motor impairments greater than controls at diagnosis despite marked differences among the clusters in cognitive and behavioral performance. Thus, while the traditional motor diagnosis selects for the identification of a predominantly motor phenotype, a multidimensional diagnosis may identify predominantly non-motor presentations.

It is unclear why certain participants were given a multidimensional diagnosis in the absence of significant impairment in cognition or behavior in cluster 3 (cognitively preserved). This was not related to worse functional performance in this group. It may be that worse chorea in this group influenced raters to make a diagnosis even when the overall motor impairment was not deemed sufficient to make a motor diagnosis; or this could reflect differences in how raters diagnose HD. In the future it may be useful to ask raters what factors influenced their diagnostic decision. It may also be beneficial to establish objective methods for diagnosis, such as the establishment of certain cut-off scores on the UHDRS.

Despite these findings, even individuals receiving a multidimensional diagnosis are being identified relatively late after the accumulation of significant clinical signs. PREDICT-HD and other studies suggest that striatal atrophy and clinical features may develop decades prior to diagnosis (Aylward et al., 2000; Thieben et al., 2002; Paulsen et al., 2008). Recently, Sperling et al. published recommendations from the National Institute for Aging and the Alzheimer's Association Working Group for the research diagnosis of preclinical Alzheimer disease (AD) (Sperling et al., 2011). They proposed a staged diagnosis for preclinical AD with the earliest stage being associated with biomarkers of AD pathophysiology (A-beta on PET or in CSF), followed by biomarker evidence of neuronal injury (atrophy on MRI) and finally the presence of subtle clinical signs that did not meet criteria for mild cognitive impairment (Albert et al., 2011). Using a similar strategy in prodromal HD, many CAG expanded individuals at the time of enrollment in PREDICT-HD already had evidence of subtle motor, cognitive, and behavioral features and would have fallen into the last preclinical stage using the AD model (Solomon et al., 2007; Beglinger et al., 2008; Biglan et al., 2009; Duff et al., 2010; Stout et al., 2011).

While HD does not yet have the same breadth of valid and specific biomarkers as the AD research community, the identification of a similar staged categorization of prodromal HD could be considered using neuroimaging biomarkers. Thus in stage 1, CAG expanded individuals would have no evidence of neuronal injury using volumetric MRI imaging or clinical signs of HD on examination; in stage 2, there would be evidence of neuronal injury as suggested by striatal atrophy on volumetric MRI but no clinical signs of HD; finally in stage 3, individuals would have subtle clinical signs but would not yet meet criteria for diagnosis. Ultimately, clinical trials aimed at delaying manifestation in prodromal HD could evaluate the impact of interventions on the progression through the proposed stages, changes in volumetric imaging variables, changes in clinical measures and finally the impact on a multidimensional diagnosis of HD.

The current analysis has many limitations and caveats. Foremost is the use of different raters for the motor and multidimensional diagnoses. This introduced bias with a higher likelihood of discrepant diagnoses when the raters were different. However, different raters were relatively Uncommon (see Table 2), and there was no difference in rater type amongst the three phenotypic clusters identified. Future studies using multidimensional diagnosis should either have the rater making the diagnostic rating complete all the appropriate assessments or, if multiple individuals are doing the assessments, the multidimensional diagnosis should be based on consensus after reviewing all the data.

Another limitation was that raters were not specifically trained on how to answer Q80. Differences in the timing of diagnosis and the observed clinical phenotypes may relate to differences in how raters make the assessment of a multidimensional diagnosis. Some raters may be comfortable with diagnosing HD based on the combination of subtle motor, cognitive, and behavioral signs, whereas others may put more weight solely on the motor exam. Future studies utilizing a multidimensional diagnosis will have to standardize this decision process.

The significance of a clinical diagnosis is unclear. Striatal atrophy and subtle clinical features develop decades before traditional diagnosis. In addition, while subjects at diagnosis were more functionally impaired compared with controls, most individuals continued to work full-time and have minimal functional impairment by the measures used in this study even at the time of diagnosis. It remains to be seen whether regulatory bodies will consider a delay in diagnosis as sufficient to show that an intervention is effective or whether it will be necessary to show a slowing in functional decline. If the latter proves to be true, more refined measures of function in prodromal HD will be necessary.

Finally, it is important to emphasize that the proposed diagnostic criteria is for research purposes only and not necessarily for the clinical diagnosis of patients. The decision to render a clinical diagnosis in individuals at risk for HD is a complicated one based on clinical features of disease, patient preferences, and a detailed understanding of relevant psychosocial factors. The potential clinical and emotional impact on patients and their families of diagnosing individuals earlier and with less motor impairment remains unknown.

A multidimensional diagnosis occurs earlier and with less motor and cognitive impairment than the traditional motor diagnosis and identifies clinical phenotypes that may have predominant non-motor features. A staging system in prodromal HD, similar to that proposed in AD, may be of value. A better understanding of diagnostic decision making may allow for better standardization of diagnosis, and the development of clear criteria for research and clinical diagnoses that may be utilized as an outcome measure in future trials aimed at delaying diagnosis in prodromal HD.

### Conflict of interest statement

Dr. Biglan receives grant funding from the MJFF, NINDS, NPF, HDSA, Lundbeck, Google Inc., Marvell Inc., Excellus-Blue Cross Consultant for Theravance, and Lundbeck; he also has contracts with Presbyterian Home for Central NY and the Parkinson Support Group for Central NY, and Susquehanna Nursing Home and Rehabilitation Center. Dr. Geschwind does consulting for Medacoro Consultant, Clinical Advisors, and Gerson Lehman Group and is on the Editorial Board of Dementia Cognition. Dr. Raymond's research is supported by operating grants from the Canadian Institutes of Research, the Cure Huntington Disease Initiative Foundation, Inc., and the Michael J Fox Foundation. Dr. Testa received research support from the Huntington Society of Canada (principal investigator), as well as the Tremor Research Group, HighQ Foundation, and Huntington Study Group (site principal investigator subcontracts). The Tremor Research Group subcontract sponsor is GlaxoSmithKline. Huntington Study Group subcontract sponsors are NIH/NINDS or Medivation. She was a co-investigator on NIH/NCRR 2R24RR018827-05A1. Dr. Wojcieszek receives clinical trial funding from Medivation, Novartis, Scheering Plough, and TEVA; she is also part of the TEVA speaker's bureau. All other authors report no disclosures or potential conflicts of interest.

## Supplementary material

Table e-1 **Functional status by clusters**.

Table e-2 **Proportion of same versus different raters by cluster^*^**.

## Abbreviations

AD: Alzheimer disease
CAG: cytosine-adenine-guanine
DCL: diagnostic confidence level
HD: Huntington disease
PREDICT-HD: Neurobiological Predictors of Huntington's Disease
Q80: Question 80
SDM: symbol digit modalities
TFC: Total Functional Capacity
UHDRS: Unified Huntington Disease Rating Scale.
