# Supplementary material for: Refining the diagnosis of Huntington disease: the PREDICT-HD study
Source: Front Aging Neurosci. 2013 Apr 2;5:12. doi: 10.3389/fnagi.2013.00012 (PMC3613616; doi:10.3389/fnagi.2013.00012)
Supplement: Table e-1 — Functional status by clusters. [file 42536_Paulsen_DataSheet1.DOCX]

**Table e-1 Functional Status by Clusters**

| **Clinical Variables** | **Control**  **(n=194)** | **Cluster 1 (n=23)** | **Cluster 2 (n=14)** | **Cluster 3 (n=31)** | **Fisher exact**  ***p*-value** | **Pair-wise Comparisons**  **(alpha≤0.01)** |
| --- | --- | --- | --- | --- | --- | --- |
| **TFC (%<11)** | 0 | 21.74 | 21.43 | 6.45 | <0.0001 | C1,C2,C3>Control |
| **% employable accustomed work*** | 97.94 | 73.91 | 78.57 | 87.10 | <0.0001 | Control>C1,C2,C3 |
| **% employable**  **any work^φ^** | 98.45 | 78.26 | 85.71 | 93.55 | <0.0001 | Control>C1 |

*Question 43 of the UHDRS Functional Assessment Check List

^φ^Question 44 of the UHDRS Functional Assessment Check List

Cluster 1 = predominantly cognitive

Cluster 2 = predominantly behavioral

Cluster 3 = cognitively preserved
