# Supplementary material for: Refining the diagnosis of Huntington disease: the PREDICT-HD study
Source: Front Aging Neurosci. 2013 Apr 2;5:12. doi: 10.3389/fnagi.2013.00012 (PMC3613616; doi:10.3389/fnagi.2013.00012)
Supplement: Table e-2 — Proportion of same versus different raters by cluster*. [file 42536_Paulsen_DataSheet2.DOCX]

**Table e-2 Proportion of same versus different raters by cluster ***

| **Rater Type** | **Cluster 1 (n=23)** | **Cluster 2 (n=14)** | **Cluster 3 (n=31)** | **Total** |
| --- | --- | --- | --- | --- |
| **Same rater** | 7 (30.4%) | 3 (21.4%) | 8 (25.8%) | 18 |
| **Different rater** | 16 (69.6%) | 11 (78.6%) | 23 (74.2%) | 50 |

**p*-value = 0.48 for comparison of cluster by rater type

Cluster 1 = predominantly cognitive

Cluster 2 = predominantly behavioral

Cluster 3 = cognitively preserved
